# Ultralow Strain‐Induced Emergent Polarization Structures in a Flexible Freestanding BaTiO_3_ Membrane

**DOI:** 10.1002/advs.202401657

**Published:** 2024-04-22

**Authors:** Jie Wang, Zhen Liu, Qixiang Wang, Fang Nie, Yanan Chen, Gang Tian, Hong Fang, Bin He, Jinrui Guo, Limei Zheng, Changjian Li, Weiming Lü, Shishen Yan

**Affiliations:** ^1^ Spintronics Institute School of Physics and Technology University of Jinan Jinan 250022 China; ^2^ Functional Materials and Acousto‐Optic Instruments Institute School of Instrumentation Science and Engineering Harbin Institute of Technology Harbin 150080 China; ^3^ School of Physics State Key Laboratory of Crystal Materials Shandong University Jinan 250100 China; ^4^ School of Materials Science and Engineering Nanjing University of Science and Technology Nanjing 210094 China; ^5^ Department of Materials Science and Engineering Southern University of Science and Technology Shenzhen Guangdong 518055 China; ^6^ Guangdong Provincial Key Laboratory of Functional Oxide Materials and Devices Southern University of Science and Technology Shenzhen Guangdong 518055 China

**Keywords:** freestanding membrane, polarization, strain engineering, vortex, zigzag morphology

## Abstract

The engineering of ferroic orders, which involves the evolution of atomic structure and local ferroic configuration in the development of next‐generation electronic devices. Until now, diverse polarization structures and topological domains are obtained in ferroelectric thin films or heterostructures, and the polarization switching and subsequent domain nucleation are found to be more conducive to building energy‐efficient and multifunctional polarization structures. In this work, a continuous and periodic strain in a flexible freestanding BaTiO_3_ membrane to achieve a zigzag morphology is introduced. The polar head/tail boundaries and vortex/anti‐vortex domains are constructed by a compressive strain as low as ≈0.5%, which is extremely lower than that used in epitaxial rigid ferroelectrics. Overall, this study c efficient polarization structures, which is of both theoretical value and practical significance for the development of next‐generation flexible multifunctional devices.

## Graphical abstract

To overcome the limitations of conventional rigid epitaxial thin films and heterostructures, a flexible single‐crystal freestanding BaTiO_3_ membrane to construct a continuous uniaxial/biaxial compressive strain distribution with a zigzag morphology is employed. The polar head/tail boundaries and vortex/anti‐vortex are demonstrated at the wrinkle peak/valley and joint morphologies of BTO, which are interestingly driven by ultralow uniaxial and biaxial strains of ≈0.5%.

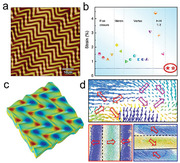

## 1 Introduction

Over the recent years, the development of novel polarization structures, such as flux‐closure structures,^[^ ^1^, ^2^ ^]^ skyrmion,^[^ ^3^, ^4^ ^]^ meron,^[^ ^5^ ^]^ vortex,^[^ ^6^, ^7^, ^8^ ^]^ etc.,^[^ ^9^, ^10^, ^11^, ^12^, ^13^ ^]^ in ferroelectric thin films and heterostructures has garnered extensive research interest. The exotic polar ordering can not only enrich the fundamental understanding of condensed matter physics but also hold great promise for nanoscale electronic applications. These novel structural topologies leading to non‐bulk emergent phenomena arise due to the competition between various types of energies, such as gradient, elastic, and electrostatic energies, which can be engineered via a particular misfit strain, charge screening, and/or external electric field.^[^ ^4^, ^14^, ^15^, ^16^, ^17^ ^]^ At the application level, ferroelectric switching can be employed in random access memory since the polarization orientations are nonvolatile and detectable.^[^ ^18^ ^]^ The development of ferroelectric domains, such as the flux‐closure domains, vortex, etc., is desirable for high‐density memory, mechanical sensors, and transducers.^[^ ^19^, ^20^, ^21^ ^]^ However, an effective and clear strategy for designing/modulating a specific ferroelectric polar structure has not been established yet.

In this study, to achieve the evolution of polarization configuration by curvature change and fabricate desirable polarization structures, a continuous and periodic strain is applied to a freestanding BaTiO_3_ (BTO) membrane for obtaining a zigzag morphology. The experimentally obtained 3D piezoelectric response reveals long‐range coherent and periodic polarization distributions in the structure. The polar head/tail boundaries and vortex/anti‐vortex are demonstrated at the wrinkle peak/valley and joints of BTO. Remarkably, these features are driven by ultralow uniaxial and biaxial strains (≈0.5%).

## 2 Results and Discussion

**Figure 1:**
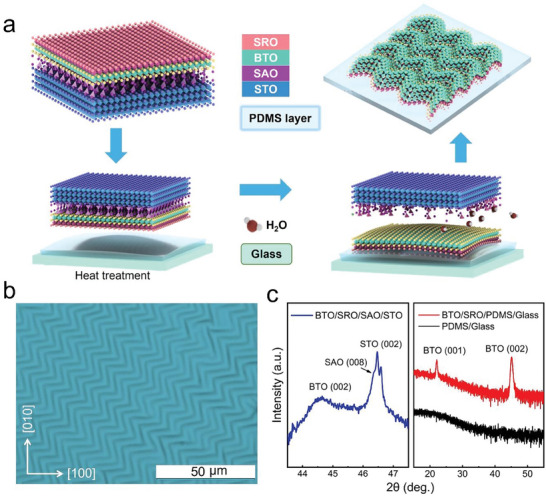
Fabrication of BTO film with zigzag‐shaped wrinkles. a) Schematic of the fabrication process of a zigzag‐patterned wrinkled BTO film. b) Optical microscopy images of zigzag‐wrinkled BTO film. c) XRD patterns of the BTO/SRO/SAO/STO heterostructure and the freestanding BTO/SRO heterostructure transferred onto PDMS/glass.

The freestanding zigzag BTO membrane was obtained by the water‐solvation method from a rigid epitaxial SrRuO_3_/BTO/Sr_3_Al_2_O_6_/SrTiO_3_ (SRO/BTO/SAO/STO) heterostructure, where SRO, SAO, and STO were used as the electrode, water‐soluble interlayer, and substrate, respectively. For obtaining the freestanding BTO membrane, the SRO/BTO/SAO/STO heterostructure was immersed in deionized water, and then it was transferred onto a polydimethylsiloxane (PDMS) substrate. A schematic of the preparation process is shown in **Figure** **1**a. The water‐solvation method and transfer technology enable us to manipulate the freestanding BTO membrane to the expected morphology while also facilitating its integration with other functional materials (e.g., silicon, organic polymers, metal/alloy). The preheated PDMS carrier underwent significant volume contraction during the cooling process, and the adhesive freestanding BTO membrane was subjected to a biaxial strain, resulting in a zigzag topography (see Experimental Section). Different from conventional strain engineering in epitaxial heterostructures, which are rigid and do not allow continuous strain tunability, flexible zigzag BTO membrane exhibits continuous and periodic morphological changes via the curvature transform in wrinkles and joints.

Figure 1b shows an optical microscopy image of the freestanding wrinkled BTO membrane. The periodic distribution of zigzag‐shaped wrinkles over a large area is clear, with each wrinkle swerving at ≈90° every 10 to 15 µm. X‐ray diffraction (XRD) was employed to analyze the crystal structures of the rigid and freestanding BTO heterostructures. As illustrated in Figure 1c, the BTO layer in the BTO/SRO/SAO/STO structure grows along the (001) direction, without any notable impurity or structural phases. Post the freestanding process, process, only BTO peaks are observed. Further examinations reveal that the out‐of‐plane (OOP) lattice constant of the freestanding BTO membrane is ≈4.016 Å, which is smaller than that of the rigid heterostructure (≈4.064 Å). This phenomenon indicates the release of in‐plane (IP) compressive strain.

**Figure 2:**
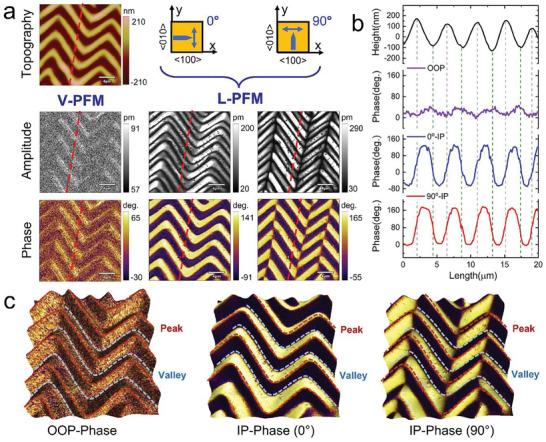
Domain structures of zigzag‐wrinkled BTO film. a) Topographic image of wrinkled BTO film, giving rise to zigzag pattern. V‐PFM and L‐PFM amplitude and phase images for two different sample rotation angles of 0° and 90°. b) Line profiles of the height, OOP phase, and IP phase (0° and 90°) data (average over 6 pixels) along the red dotted lines in (a). c) Typical OOP and IP phase images overlapped on 3D morphology. The red and blue dotted curves indicate the position of the peak and valley, respectively.

Understanding the 3D polarization configuration of a wrinkled BTO membrane is crucial for revealing the relationship between the polarization structure and strain distribution. For the wrinkled BTO film, the average distance between the peak and valley is ≈2.7 µm, and the average peak height is ≈290 nm. To evaluate the polarization configuration, piezoresponse force microscopy (PFM) was employed to obtain the piezoresponse under both vertical and lateral modes (referred to as V‐PFM and L‐PFM, respectively), and the results are shown in **Figure** **2**a. Specifically, the L‐PFM measurements were conducted along both (010)‐ and (100)‐directions, denoted as 0°‐IP and 90°‐IP, respectively. There are significant differences between the 0°‐IP and 90°‐IP measurement results. In particular, the IP component strongly depends on the morphology. This stark contrast indicates the occurrence of periodic phase development and the presence of domain walls separating opposing domain structures. The V‐PFM images show much weaker topography dependence. The magnified V‐PFM images of the peak and valley regions reveal the presence of multiple nanodomains (Figure S1, Supporting Information), indicating relatively disordered polarization patterns and ferroelectric domains perpendicular to the film surface. To further elucidate the peculiarity of the polarization structures in the freestanding wrinkled BTO membrane, the linear features of V‐PFM and L‐PFM are summarized in Figure 2b and Figure S2 (Supporting Information) based on the red dashed line in Figure 2a. The periodic wrinkles in the BTO film have a significant effect on the 0°‐IP and 90°‐IP phases, resulting in a “braided” ferroelectric superstructure. This braided domain structure indicates an opposite IP polarization orientation between the two sides of the highest peak and lowest valley locations as well as between the two sides of the wrinkle inflection points. Additionally, the transition regions between the peaks and valleys show an enhanced IP piezoresponse, while the valley regions exhibit a strong piezoresponse in the OOP direction (Figure S2a,b, Supporting Information). The distinct polarization states of the peaks and valleys are also evident in the surface potential distribution (Figure S3, Supporting Information). Figure 2c displays the 3D representations of the phases obtained from V‐PFM and L‐PFM measurements. This complicated polarization structure in our ferroelectric as a function of morphological variation cannot be easily obtained in rigid bulk materials or epitaxial thin films. These findings significantly contribute to the fundamental understanding of polarization structures and ferroelectric domains under non‐uniformly distributed stress.

To ensure the experimental accuracy, we tested additional samples and obtained PFM data at different rotation angles on a larger scale. The results exhibit a high level of consistency, as illustrated in Figure S4 (Supporting Information). Furthermore, PFM spectroscopic measurements were conducted to investigate the domain‐switching dynamics of the wrinkled BTO film. The representative amplitude and phase hysteresis loops of peak, valley, and transition regions are shown in Figure S5 (Supporting Information). The ≈180° change in the phase loops clearly demonstrates the polarization switching in the different regions. The hysteresis loop in the valley region shows an obvious negative mark (−3.1 V), while the peak region shows a much smaller negative signal (−1.1 V). The transition region (nearly strain‐free region) shows a rather symmetric loop with negligible imprint.

**Figure 3:**
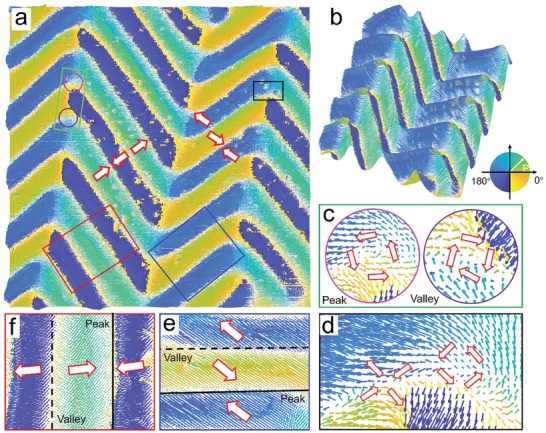
Conversion of V‐PFM and L‐PFM images of the wrinkled BTO films into vector contours based on MATLAB analysis. a) and b) 2D polarization vector contours and 3D vector map of 15  ×  15 µm^2^ region, respectively. c) and d) Magnified images of green and black square areas in a), respectively. c) Vortex pair and d) vortex–antivortex pair. e) and f) Magnified images of red and blue square areas in a). The long‐range ordered and periodic H–H and T–T ferroelectric domain structures are observed in the peak and valley regions, respectively.

By utilizing the function A × cos(θ) (where A represents the amplitude and θ denotes the phase angle), the amplitude and phase can be transformed into PFM piezoresponse signal contours.^[^ ^22^, ^23^, ^24^ ^]^ By integrating L‐PFM and V‐PFM measurement results, the 3D components of polarization in the wrinkled BTO film can be determined. Consequently, a comprehensive spatial distribution of the polarization vector can be determined through MATLAB software (see details in Experimental section). The PFM images of x and y components obtained by 0° and 90° L‐PFM are converted into 2D vector contours, reflecting the local polarization distributions of the IP components, as shown in **Figure** **3**a. In Figure 3b and Figure S7 (Supporting Information), the z component (V‐PFM) data is superimposed to construct a 3D vector profile, which clearly illustrates the significant influence of BTO morphology on the periodic polarization. Here, the arrows of different colors indicate the polarization rotation. According to these contours, it is inferred that the wrinkled BTO membrane has a specific domain structure. Vortex pairs (close to 1 µm in diameter) and vortex–antivortex pairs are observed at the wrinkle inflection points, as shown in Figure 3c,d, and Figure S6 (Supporting Information). In addition, long‐range ordered and periodic head‐to‐head (H–H) and tail‐to‐tail (T–T) ferroelectric domain structures are observed, as depicted in Figure 3e,f. Remarkably, these specific domain patterns spontaneously emerge in the freestanding wrinkled BTO membrane, suggesting that they are likely the most stable states, which is crucial for further manipulation.

The observed topological domains are in stark contrast to the previously reported a/c mixed domains in freestanding BTO films because the polarization of the domains deviates from the predetermined 90° and 180° directions. These domains may be formed due to the competition among gradient energy, elastic energy, and electrostatic energy, all of which can greatly change the local anisotropy, thereby changing the polarization distribution. The gradient energy and electrostatic energy are reduced by the rotation of the electric dipole to balance the increased elastic energy due to the zigzag pattern, minimizing the minimum local total energy required for the formation of vortex domains. Additionally, the observed charge domain cores in an H–H or T–T configuration bear some resemblance to the charge domain walls, which can be stabilized by charge accumulation from internal electrons, holes, and ionic defects.^[^ ^25^, ^26^, ^27^, ^28^ ^]^ The free electrons from the ambient air can also be injected onto the surface close to the charge domain walls/cores, reducing the formation energy.^[^ ^29^ ^]^

Considering the significant role of strain engineering in regulating the ferroelectric domains, it is imperative to quantify the strain in the freestanding wrinkled films. We performed statistical measurements of the wavelength *λ* (half of the peak‐to‐peak (or valley‐to‐valley) distance) and height *h* (half of the peak‐to‐valley distance) of multiple wrinkles.

The radius of curvature can be obtained as follows:

(1) $$R=\;\frac{\lambda^{2}}{8h}\;+\;\frac{h}{2}$$

Therefore, the bending strain of the film can be estimated as^[^ ^30^, ^31^ ^]^

(2) $$\varepsilon \;=\;\frac{t}{2R}$$

where *t* is the film thickness (22 nm).

**Figure 4:**
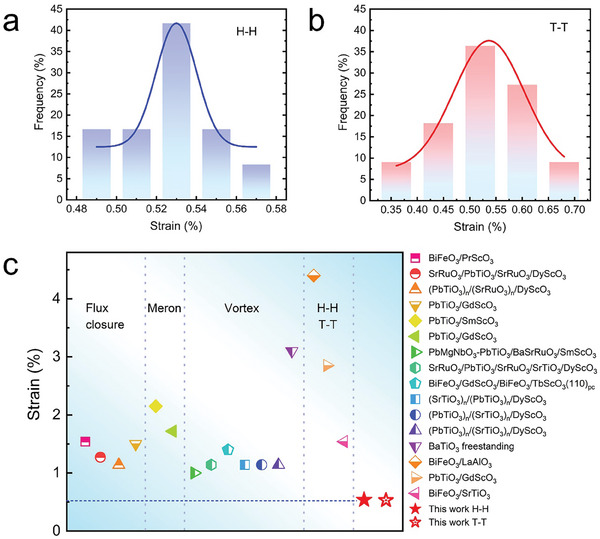
Bending strain values of BTO film. Statistical histograms of strain in H–H a) and T–T b) regions. c) Strain values reported in recent years that resulted in ferroelectric topological domains.^[^ ^1^, ^5^, ^6^, ^13^, ^32^, ^33^, ^34^, ^35^, ^36^, ^37^, ^38^, ^39^, ^40^, ^41^, ^42^ ^]^

The average strain for the H–H and T–T structures is only 0.52%, as shown in **Figure** **4**a,b. It is worth noting that the chiral vortex states at the inflection points of the wrinkles experience biaxial strain. Moreover, the strain data from recent studies on ferroelectric thin films exhibiting singular polarization structures are summarized in Figure 4c. Interestingly, the proposed BTO film exhibits the lowest strain.

**Figure 5:**
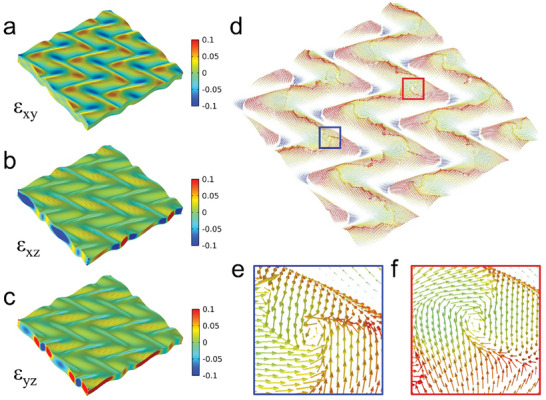
Phase field simulation of the zigzag‐wrinkled BTO film. Distribution of shear strain components: a) ε_xy_, b) ε_xz_, and c) ε_yz_. d) 3D polarization vector contour superimposed on the topography. e) and f) Magnified images of the blue and red square areas in d), respectively. Vortex states in counterclockwise e) and clockwise f) directions are observed.

Next, phase‐field simulations were employed to further understand the formation mechanism of ferroelectric domains due to the superimposed strain from the zigzag‐wrinkled morphology. The OOP elastic displacement of the wrinkled film is $U_{z}=U_{0}\cos\left\{\frac{2\pi }{L}\left(x-y\right)+2\cos[\frac{2\pi }{L}\left(x+y)\right]\right\}$, where the amplitude *U_0_* is estimated to be ≈100 nm and the period length *L* ≈8 µm according to the PFM measurement in Figure 2. The simulated polarization arrangement and elastic strain distribution of the top surface are shown in **Figure** **5**. Periodic H–H domain structures are observed at the peak, and vortex states exist at the inflection region of wrinkles (Figure 5d–f), which are in good agreement with the experimental results in Figure 3. The formation of topological polar vortices can be attributed to the large shear strain component ε_xy_ at the inflection region of wrinkles, which causes IP deviation of the polarization direction (See Figure 5a–c). We further investigated the evolution of the domain structure by modifying the amplitude of elastic displacement and the period of wrinkles in the simulation, and the results are shown in Figures S9 and S10 (Supporting Information). Polar vortices are not observed when the period length *L* exceeds 15 µm. As the period length decreases, the OOP strain distribution becomes more complex, and the strain becomes more focused at the zigzag inflection point. The simple a/c domain distribution with lower energy evolves into an H–H polarization structure with higher energy, and the vortex state appears. On the other hand, as the amplitude of wrinkles increases, the strain gradually intensifies, leading to an increase in the number of polar vortices. These results indicate that desired polarization states can be obtained by the engineered wrinkled morphology.

**Figure 6:**
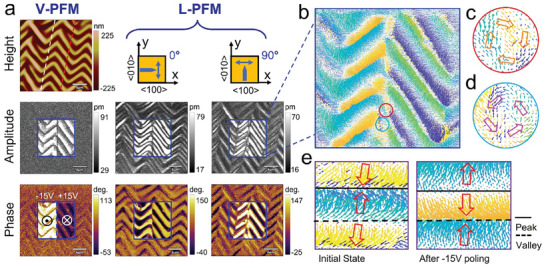
Electric switching behavior of zigzag‐wrinkled BTO film. a) V‐PFM and L‐PFM (0° and 90°) images after poling with −15 V (left box) and 15 V (right box). b) 2D direction map of the domain region. c) and d) Magnified images of red and light blue boxes in b); vortex domains rotating clockwise c) and counterclockwise d) are observed. e) The peak region changes from the H–H structure in the initial state to the T–T structure, while the valley shows the opposite change.

Furthermore, to investigate the tunability of vortex domains and the response of periodic domain structures to external electric field stimuli, we applied a scanning tip bias of ±15 V to a 15 × 7.5 µm^2^ region of the wrinkled BTO film. The OOP‐ and IP‐PFM amplitude and phase images (0° and 90°) have been acquired under an AC amplitude of 500 mV, following the application of ±5 V DC voltage (**Figure** **6**a), which indicates the non‐volatile switching behavior of domains. Figure S11 (Supporting Information) provides the polarization information for the initial state of this region. Within the poled region, opposite PFM phase polarity is observed, and the PFM amplitude drops at the domain walls, which is consistent with the expected 180° polarization reversal in classical ferroelectrics. At the applied voltage, the OOP polarization direction in the peak and valley regions is opposite. Notably, the IP polarization in the peak and valley regions exhibits different responses to voltage, clearly revealing the domain switching and the “braided” structure of the IP domain pattern. Figure S11 (Supporting Information) shows the line profiles of the height, amplitude, and phase images along the white and red dotted line in Figure 6a, including PFM information of the corresponding position before applying the voltage (Figure S12, Supporting Information). Under the applied voltage, the OOP piezoresponse in the valley region is enhanced. As for the IP piezoresponse, the transition region between the peak and valley regions also exhibits obvious enhancement. Additionally, Figure 6b presents a 2D direction map of the written domain region. Vortex domains with clockwise or counterclockwise rotation are observed at the transition of positive and negative voltages, as shown in Figure 6c,d. These results indicate that vortex domains can be both spontaneously generated due to strain and generated at any position through electric field manipulation in the freestanding wrinkled BTO films. Meanwhile, under the influence of negative voltage, the peak regions transition from the H–H structure in the initial state to the T–T structure, while the valley regions undergo the opposite change (Figure 6e). We evaluated the retention of the switched domain on the zigzag‐wrinkled BTO film and found that it remains stable even after 12 h, as shown in Figure S13 (Supporting Information). This electrically controllable behavior holds promise for application in novel flexible electronics.

## 3 Conclusion

We successfully fabricated freestanding BTO membranes with a zigzag morphology using the water‐solvation process. These films exhibited remarkable curvature‐dependent long‐range coherence and periodic distributions of polarization. Through experiments and phase‐field simulations, we observed the presence of H–H and T–T polarization boundaries as well as the formation of large‐scale chiral vortex domains. Interestingly, these singular polar structures could be induced by ultralow uniaxial and biaxial strains (≈0.5%), which is significantly lower than the previously reported values. The accumulation of charge was found to reduce the formation energy, making the singular polar structures more stable. This complicated polarization structure resulting from the morphological variation of the ferroelectric domain provides useful insights into the polarization structure and ferroelectric domain under strain engineering. The wrinkled ferroelectric oxides with different strained regions and correlated polarization distributions as well as tunable ferroelectricity can pave the way toward novel flexible electronics.

## 4 Experimental Section

### Thin Film Growth

The SRO/BTO/SAO heterostructure was grown on (001)‐oriented STO substrates by pulsed laser deposition (PLD) with a KrF (λ = 248 nm) excimer laser. The SAO layer was grown at 700 °C under an oxygen pressure of 0.05 mTorr. Subsequently, the BTO and SRO layers were deposited at 780 °C and 100 mTorr. During the deposition, the laser fluence was kept at 2.0 J cm^−2^, and the repetition rate was 2 Hz. After deposition, the samples were slowly cooled down to room temperature at the deposition pressure. The thicknesses of SRO, BTO, and SAO were 2, 20, and 20 nm. Here, the SRO layer acted as the bottom electrode for the PFM measurements.

### Preparation of Wrinkled BTO Films

PDMS prepolymer and crosslinker were mixed by weight in a certain proportion. The mixture was degassed in a vacuum desiccator for 30 min to remove residual air bubbles. Then, the treated mixture was poured into a petri dish for curing and molding. The SRO/BTO/SAO/STO heterostructures were inverted on the preheated PDMS layer at 70 °C. The sample was immersed in deionized water to etch the SAO layer, and BTO/SRO/PDMS was stripped from the STO substrate. As the sample was cooled to room temperature, the heterostructure was subjected to biaxial compressive stress due to the shrinkage of the PDMS layer, and the zigzag‐wrinkled pattern was formed.

### Piezoresponse Force Microscopy (PFM)

The polarization structures in the freestanding wrinkled BTO membrane were characterized by a commercial scanning probe microscope (MFP‐3D Origin^+^, Oxford Instruments). When the conductive probe (Arrow‐EFM, Nano World) with AC bias was in contact with the sample, the sample underwent regular expansions and contractions due to the inverse piezoelectric effect, which caused the probe to oscillate with the sample. The oscillation amplitude and phase signals were recorded, which corresponded to the piezoresponse strength and polarization orientation, respectively. Dual AC resonance tracking PFM (DART‐PFM) was used to track the shift in the contact resonance frequency caused by the surface roughness, avoid signal crosstalk, obtain more stable piezoelectric signals with higher sensitivity, and ensure the accuracy of data. The vertical deflection and torsional motion of the probe cantilever were used to detect the deformation of the sample, and the IP and OOP polarization components of the sample were obtained. To determine the domain structures, both the vertical and lateral PFM images were recorded at different sample rotation angles. The local piezoresponse hysteresis loops were measured by fixing the PFM probe on the selected position and then applying a triangular‐square waveform, accompanied with a small AC‐driven voltage from the probe.

### EFM and SKPFM Measurement

Electrostatic force microscopy (EFM) and scanning Kelvin probe force microscopy (SKPFM) are widely applied to obtain the surface potential of materials through a dual‐channel method. In the Nap mode, the first‐line scanning is used to obtain the surface morphology information of the sample, and then the probe is lifted to a certain height to detect the long‐range force (electrostatic force) signal. The operating principle of EFM can be simply interpreted as the phase difference imaging of probe vibration caused by the electrostatic force between the probe and sample. In SKPFM, a DC bias is applied to the conductive tip to balance the surface potential of the sample. The DC bias is equal to the potential difference between the tip and sample, thereby obtaining the relative surface potential distribution of the material. Therefore, EFM qualitatively reflects the potential properties of samples, and SKPFM quantifies the potential of samples.

### MATLAB Program

The relevant values were extracted from PFM topography, phase, and amplitude images using MATLAB. An (x,y,z) image was drawn with the quiver3 function in MATLAB. The formula is as follows:

(3) $$x\;=\;A_{L}\times \sin\left(\theta \right)$$

(4) $$y\;=\;A_{L}\times \cos\left(\theta \right)$$

(5) $$z\;=\;A_{V}\times \sin\left(\varphi \right)$$

where x, y, and z are the projection lengths of polarization on the a, b, and c axes; A_L_ and A_V_ represent the amplitude values of L‐PFM and V‐PFM, respectively; θ and φ represent the phase values of L‐PFM and V‐PFM, respectively.

Considering that the data measured by IP PFM are only sensitive to a single crystal axis direction, the accuracy of the spatial distribution of reconstructed polarization vectors is improved by superimposing data values from multiple angles. For example, (x+u, y+v, z) images are drawn by using the IP test results for the sample rotation angles of 0° and 90°.

(6) $$x+u\;=\;A_{L1}\times \sin\left(\theta_{1}\right)+A_{L2}\times \sin\left(\theta_{2}+90^{\circ }\right)$$

(7) $$y+v\;=\;A_{L1}\times \cos\left(\theta_{1}\right)+A_{L2}\times \cos\left(\theta_{2}+90^{\circ }\right)$$

where A_L1_, A_L2_, θ_1_, and θ_2_ are the amplitude and phase values of 0° and 90° L‐PFM, respectively.

In this way, the two sets of measurement data are related to the same coordinate axis, which eliminates the influence of rotation angle and maintains the sensitivity of the data to the two crystal axis directions. The above method can also be used to simultaneously superimpose PFM test results of multiple rotation angles to obtain a more elaborate spatial distribution of polarization.

### Phase‐Field Simulations

The total free energy *F* of ferroelectric films can be expressed as a sum of the Landau free energy *F_l_*, elastic energy *F_ela_*, electrostatic energy *F_e_*, and gradient energy *F_g_*, i.e.,

(8) $$F\;=\;F_{l}\;+\;F_{\mathrm{ela}}\;+\;F_{e}\;+\;F_{g}$$

The detailed expressions of *F_l_*, *F_ela_*, *F_e_*, and *F_g_* can be found in a previous study.^[^ ^43^ ^]^ The temporal evolution of the ferroelectric polarization is governed by the time‐dependent Landau‐Ginzburg equation:^[^ ^44^, ^45^ ^]^

(9) $$\frac{\partial P_{i}}{\partial t}\;=\;-\eta \frac{\delta F}{\delta P_{i}}$$

where η is the kinetic coefficient, and *P_i_* denotes the polarization component. The parameters of BTO are obtained from an earlier report.^[^ ^26^ ^]^ The finite element method was used for numerical analysis. Periodic boundary conditions were assumed for two IP dimensions.

To understand the influence mechanism of the wrinkles on the domain morphology, we assume an initial elastic displacement along the OOP axis of the top and bottom surfaces as follows:

(10) $$U_{z}\;=\;U_{0}\cos\left\{k_{0}(x-y)+2\cos[k_{0}\left(x+y)\right]\right\}$$

where *U*_0_ is the amplitude of the elastic displacement, and *k*_0_ =$\;\frac{2\pi }{L}\;$(*L* denotes the period of the wrinkles).

## Conflict of Interest

The authors declare no conflict of interest.

## Supporting information

Supporting Information

## Data Availability

The data that support the findings of this study are available from the corresponding author upon reasonable request.
