# Supplementary material for: Ultralow Strain‐Induced Emergent Polarization Structures in a Flexible Freestanding BaTiO3 Membrane
Source: Adv Sci (Weinh). 2024 Apr 22;11(25):2401657. doi: 10.1002/advs.202401657 (PMC11220712; doi:10.1002/advs.202401657)
Supplement: Supplementary file 1 — Supporting Information [file ADVS-11-2401657-s001.docx]

Supplementary Information

**Ultralow strain-induced emergent polarization structures in flexible freestanding BaTiO_3_ membrane**

*Jie Wang^1,2,3,†^, Zhen Liu^4,†^, Qixiang Wang^1,2^, Fang Nie^3^, Yanan Chen^1^, Gang Tian^3^, Hong Fang^1,2,3^, Bin He^1^, Jinrui Guo^1^, Limei Zheng^3,*^, Changjian Li^5,6,*^, Weiming Lü^1,2,*^, and Shishen Yan^1,3^*

*^1^* Spintronics Institute, School of Physics and Technology, University of Jinan, Jinan 250022, China.

*^2^* Functional Materials and Acousto-Optic Instruments Institute, School of Instrumentation Science and Engineering, Harbin Institute of Technology, Harbin 150080, China

*^3^* School of Physics, State Key Laboratory of Crystal Materials, Shandong University, Jinan 250100, China

*^4^* School of Materials Science and Engineering, Nanjing University of Science and Technology, Nanjing 210094, China

*^5^* Department of Materials Science and Engineering, Southern University of Science and Technology, Shenzhen 518055, Guangdong, China

*^6^* Guangdong Provincial Key Laboratory of Functional Oxide Materials and Devices, Southern University of Science and Technology, Shenzhen 518055, Guangdong, China

† These authors contributed equally to this work and should be regarded as co-first authors.

* Corresponding author.

Email address: zhenglm@sdu.edu.cn (Limei Zheng); weiminglv@hit.edu.cn (Weiming Lü).

**1: Nano-domain structure of the zigzag wrinkled BTO**


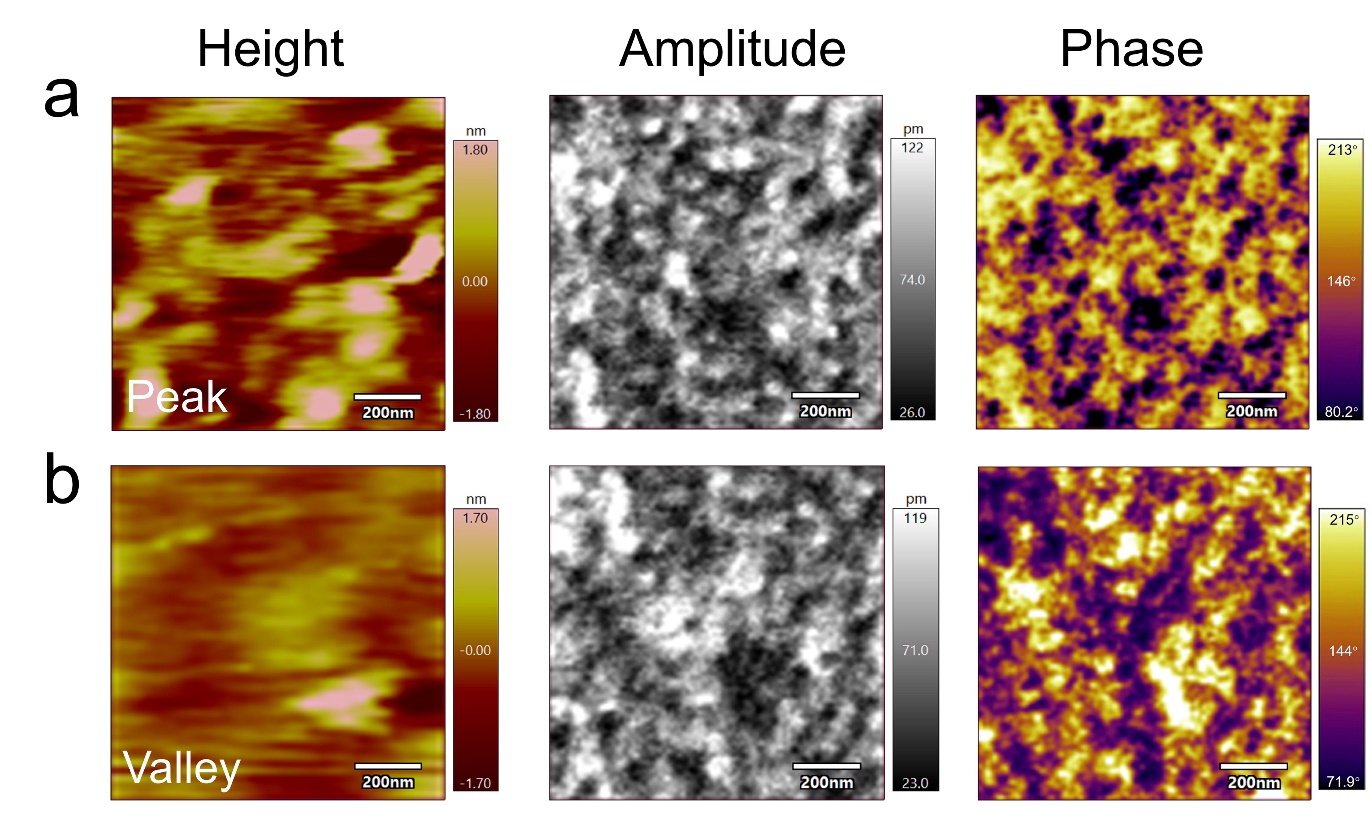


**Figure S1.** PFM measurements of nano-domains in a wrinkled BTO film. OOP-PFM images of nano-domains in peak (a) and valley (b) region. The height images were processed by flatten. Scan size is 1 μm×1 μm.

**2: Linear feature of PFM in wrinkled BTO**


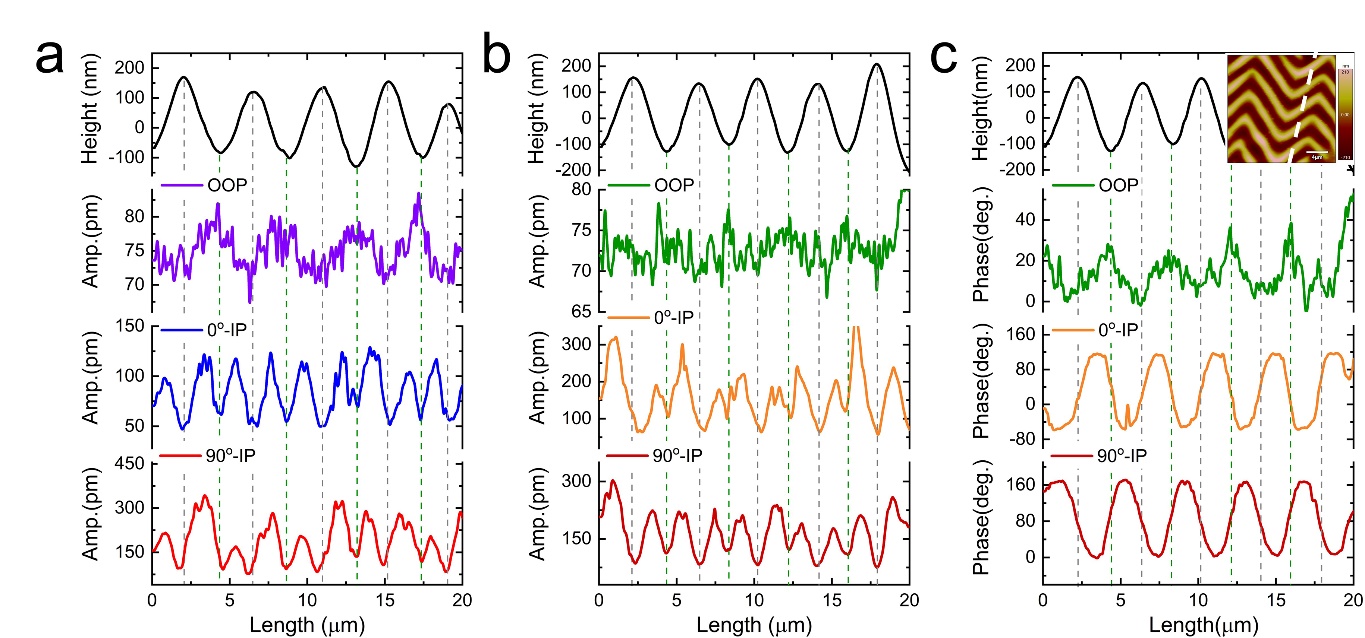


**Figure S2.** Linear feature of PFM. (a) Line profiles of the corresponding height, OOP-amplitude, IP- amplitude (0° and 90°) data (average over 6 pixels) along the red dotted lines in Figure 2a. The line profiles of the corresponding amplitude (b) and phase (c) data along the white dotted line in the illustration, and the data source is shown in Figure 2a.

**3: Surface potential distribution**


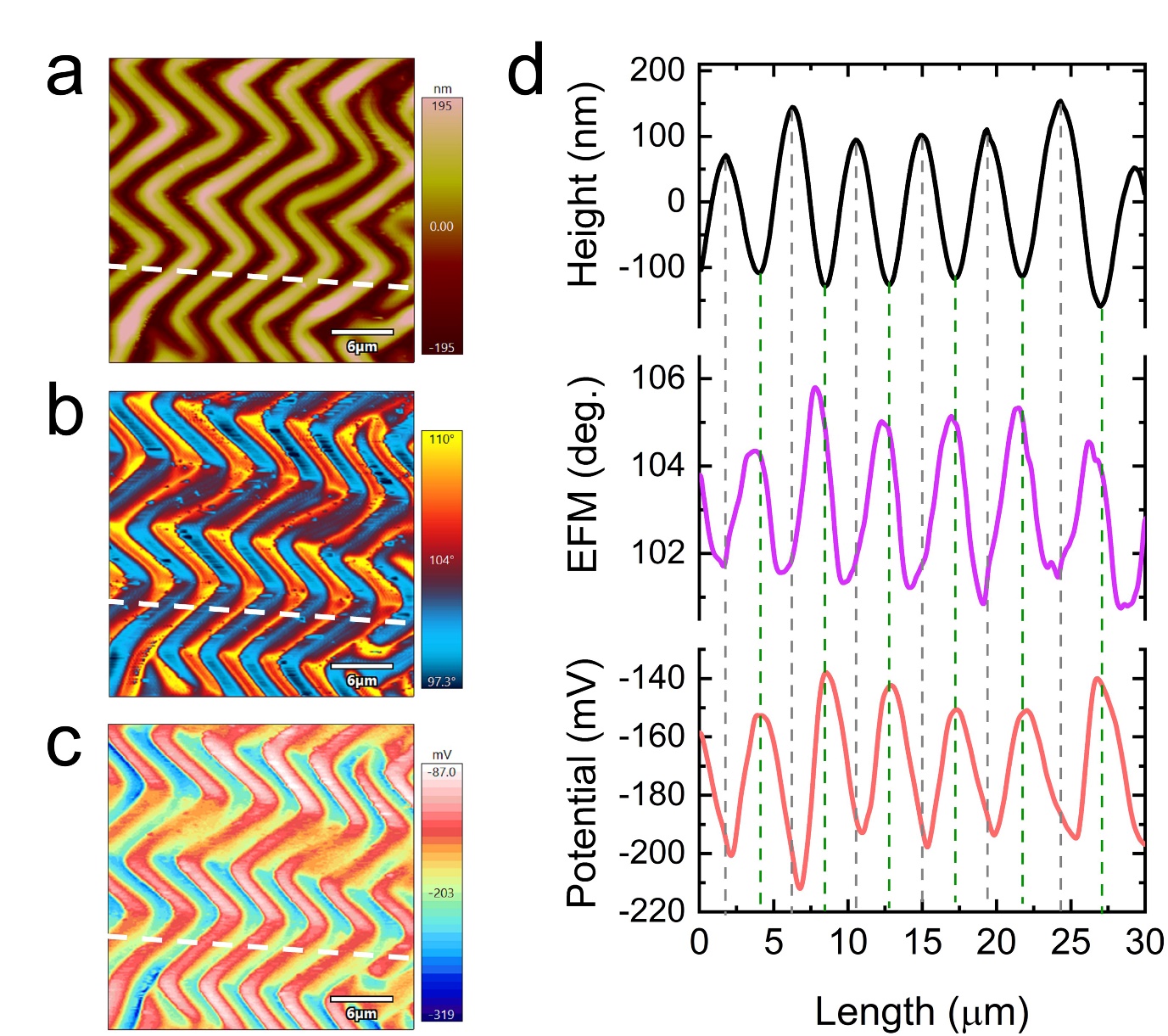


**Figure S3.** Surface potential distribution of wrinkled BTO film. (a) Height image. EFM (b) and SKPFM (c) images. (d) Line profiles of the corresponding height and surface potential along the white line. The distribution of surface potential presents a periodically waved pattern.

**4: PFM analysis of different sample rotation angles in a large range**


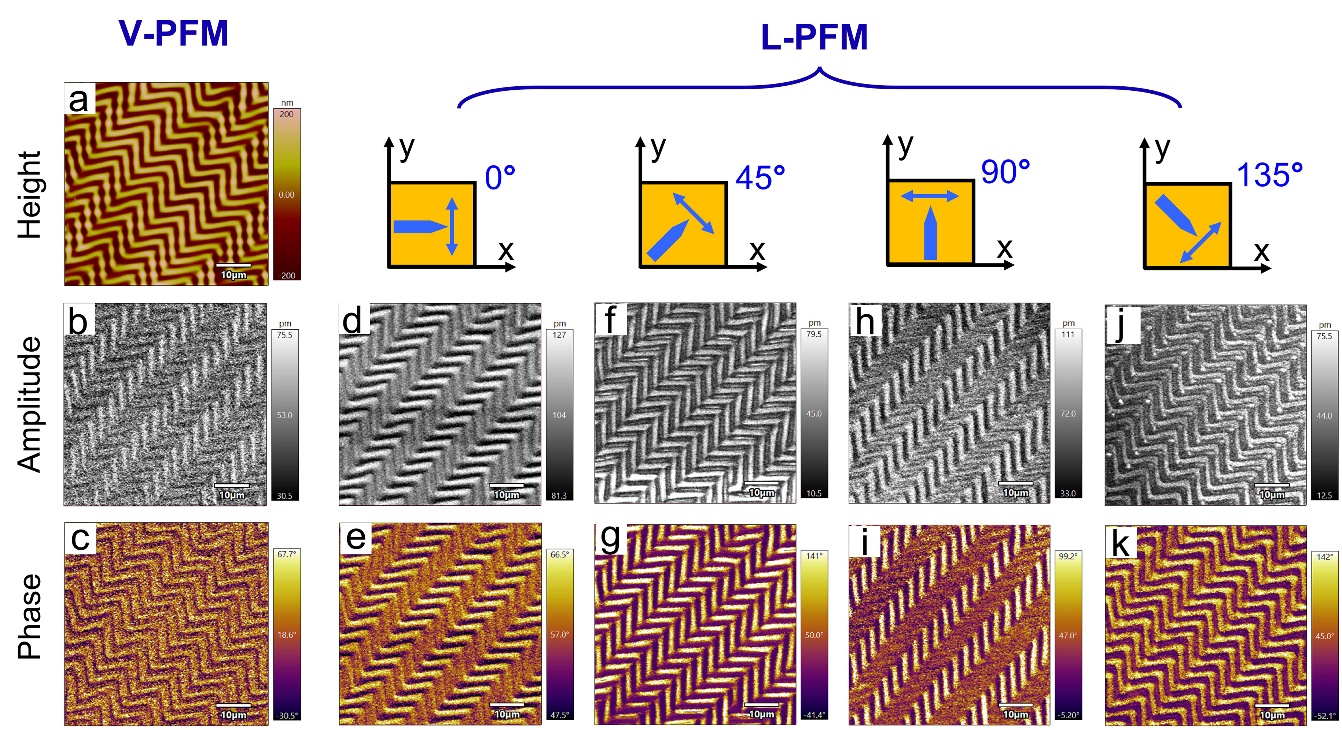


**Figure S4.** Domain structures of zigzag wrinkled BTO film in 60 μm. (a) AFM-topography image of wrinkled BTO, giving rise to zigzag pattern. (b and c) The vertical PFM (V-PFM) amplitude and phase images. (d to k) The lateral PFM (L-PFM) amplitude and phase images for four different sample rotation angles: 0º (d and e), 45º (f and h), 90º (h and i), and 135º (j and k).

**5: Local domain switching dynamics of the wrinkled BTO**


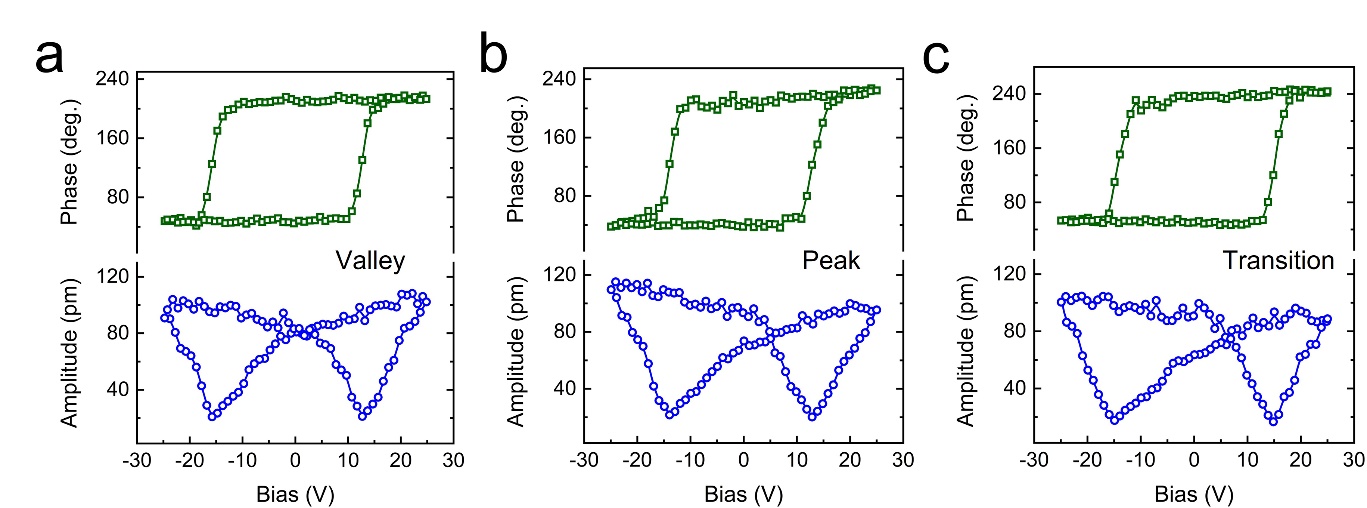


**Figure S5.** Polarization switching dynamics of the zigzag wrinkled BTO film. Typical phase and amplitude hysteresis loops observed at valley (a), peak (b), and transition (c) regions, respectively.

**6: Local vector diagrams**


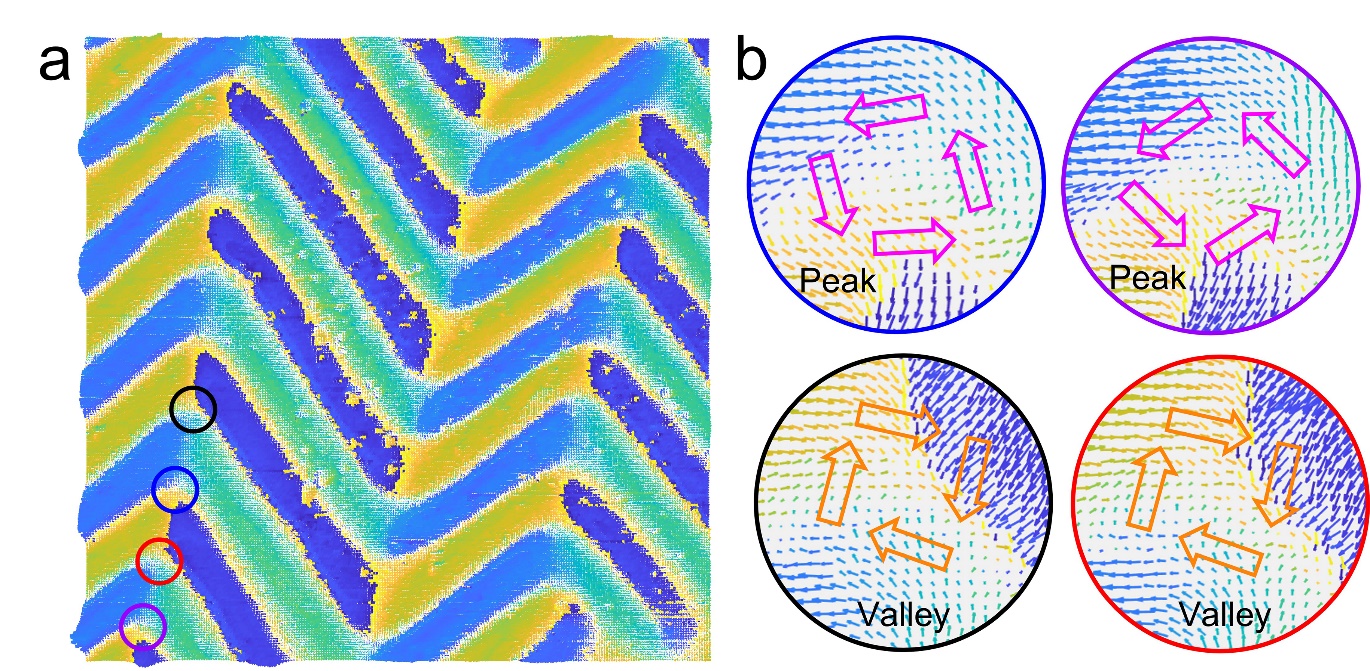


**Figure S6.** Vector map and the contours of some typical topological domains. (a) 2D polarization vector contours. (c and d) The enlarged images of the circled areas in (a). The anticlockwise and clockwise vortices were observed at the peaks and valleys, respectively.


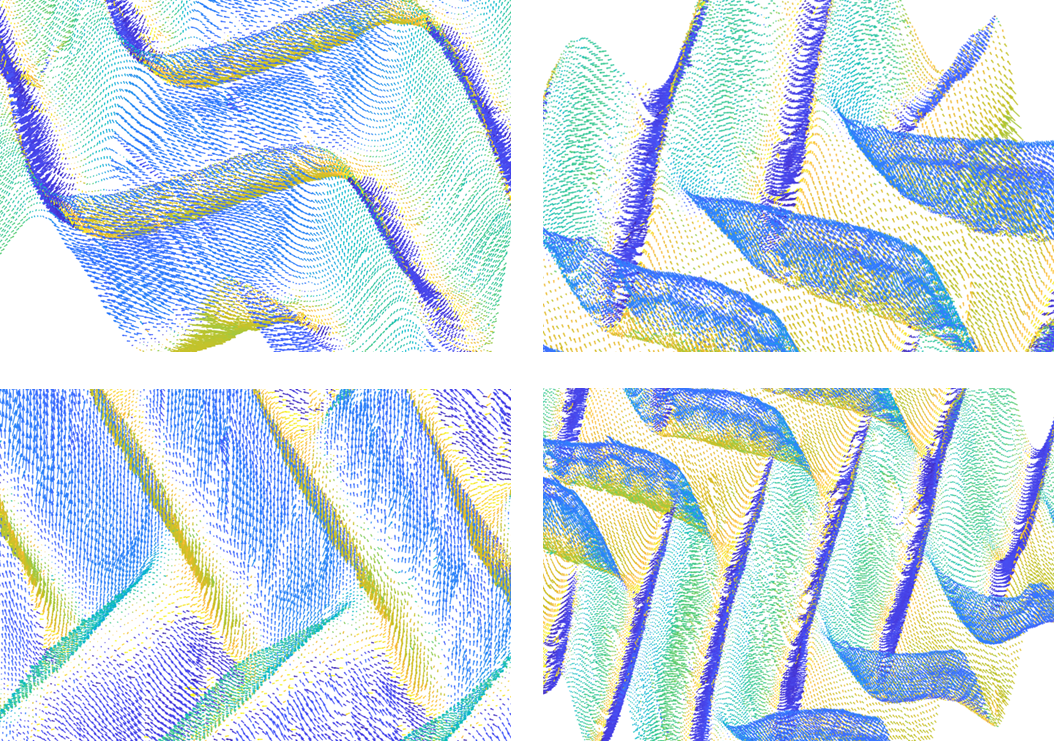


**Figure S7.** Three-dimensional vector contour magnified images of wrinkled BTO film at different angles in Figure 3b.

**7: Radius of curvature of BTO film**


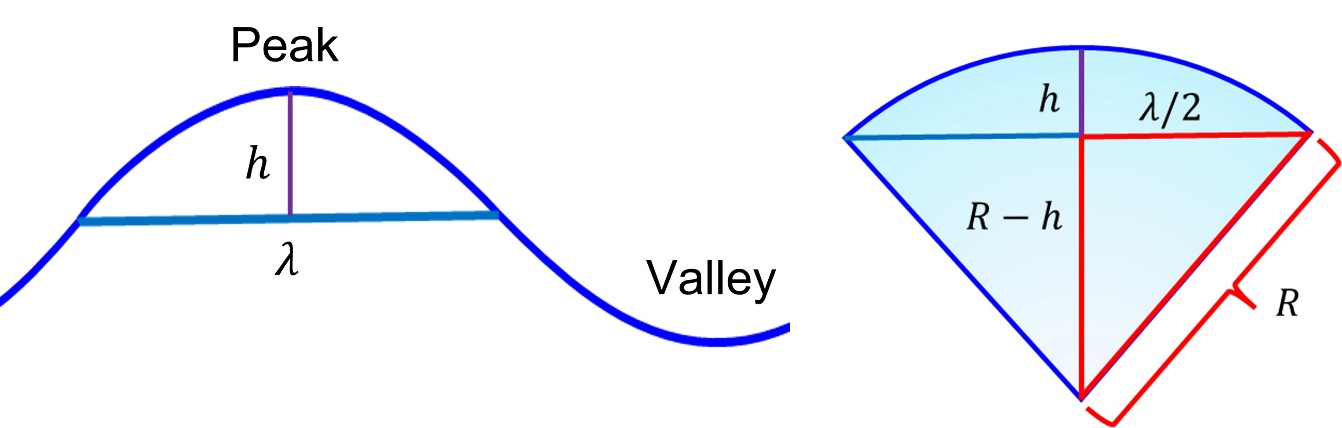


**Figure S8.** Schematic diagram of curvature radius calculation.

**8: Phase field simulation**

(1) Domain morphology with the decrease of the period *L*


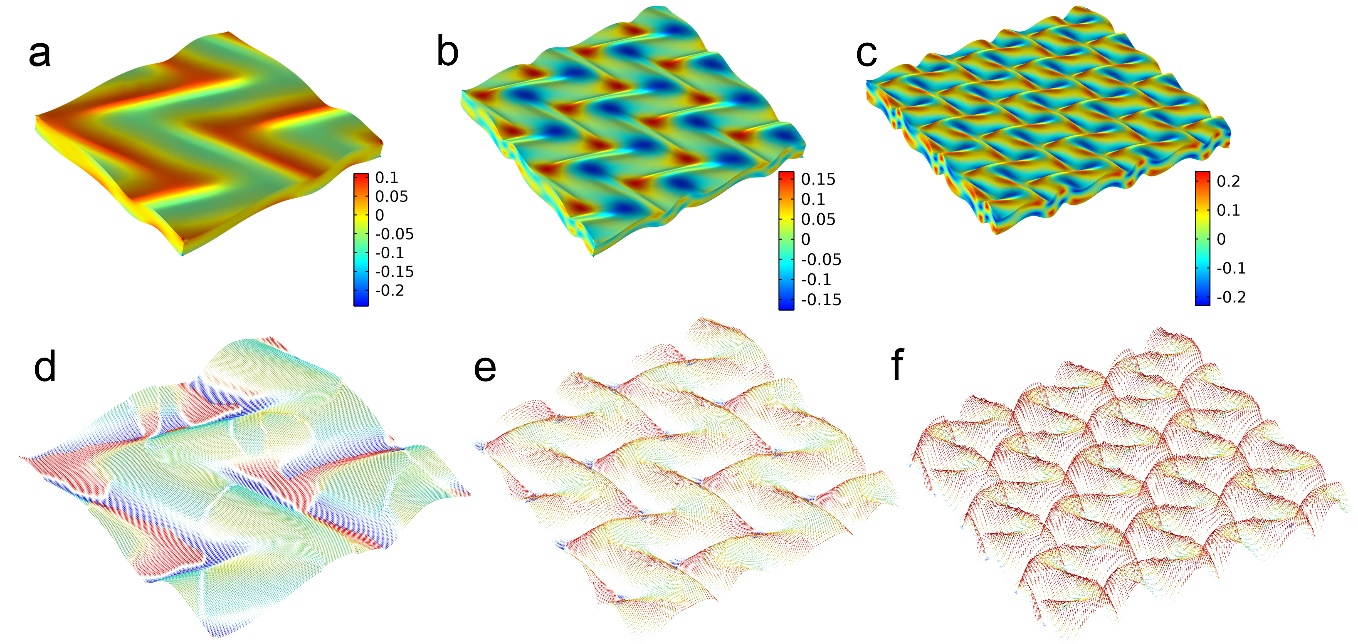


Figure S9. Simulated out-of-plane elastic strain of the wrinkle with increasing k_0_, (a) *L*=15 μm, (b) *L*=8 μm, (c) *L*=5 μm. (d)-(f) show the corresponding surface polarization distribution with decreasing *L*.

(2) Domain morphology with increasing the amplitude *U*_0_


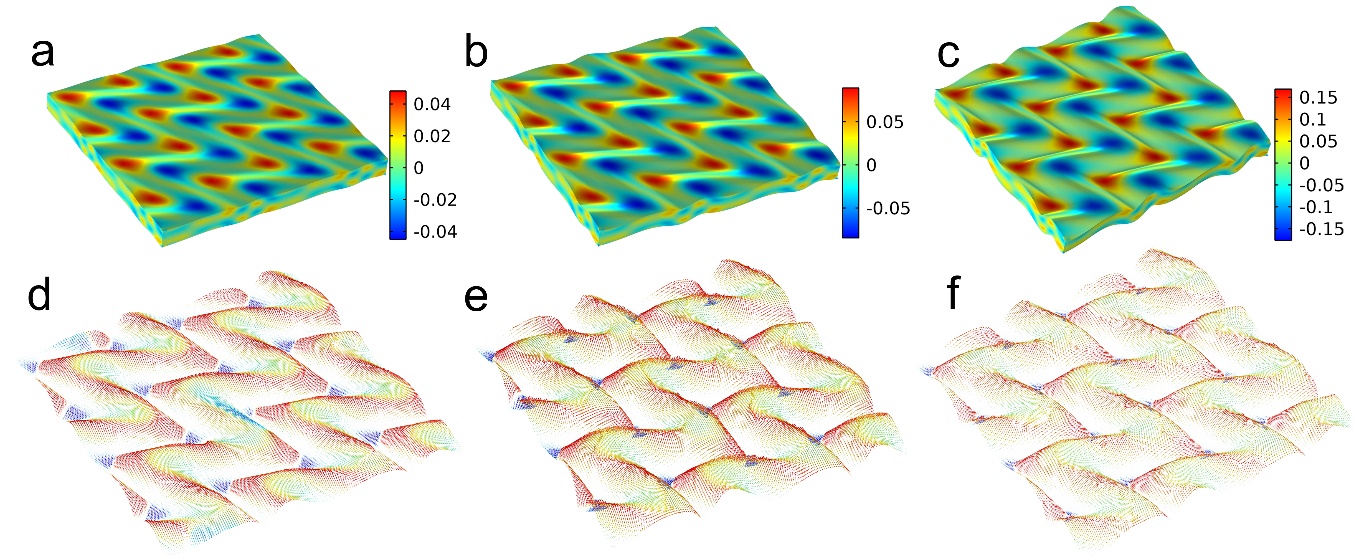


Figure S10. Simulated out-of-plane elastic strain distribution with increasing wrinkle amplitude, (a) *U*_0_=50 nm, (b) *U*_0_=100 nm, (c) *U*_0_=200 nm. (d)-(f) show the corresponding surface polarization distribution with increasing *U*_0_.

**9: PFM images and Vector map**


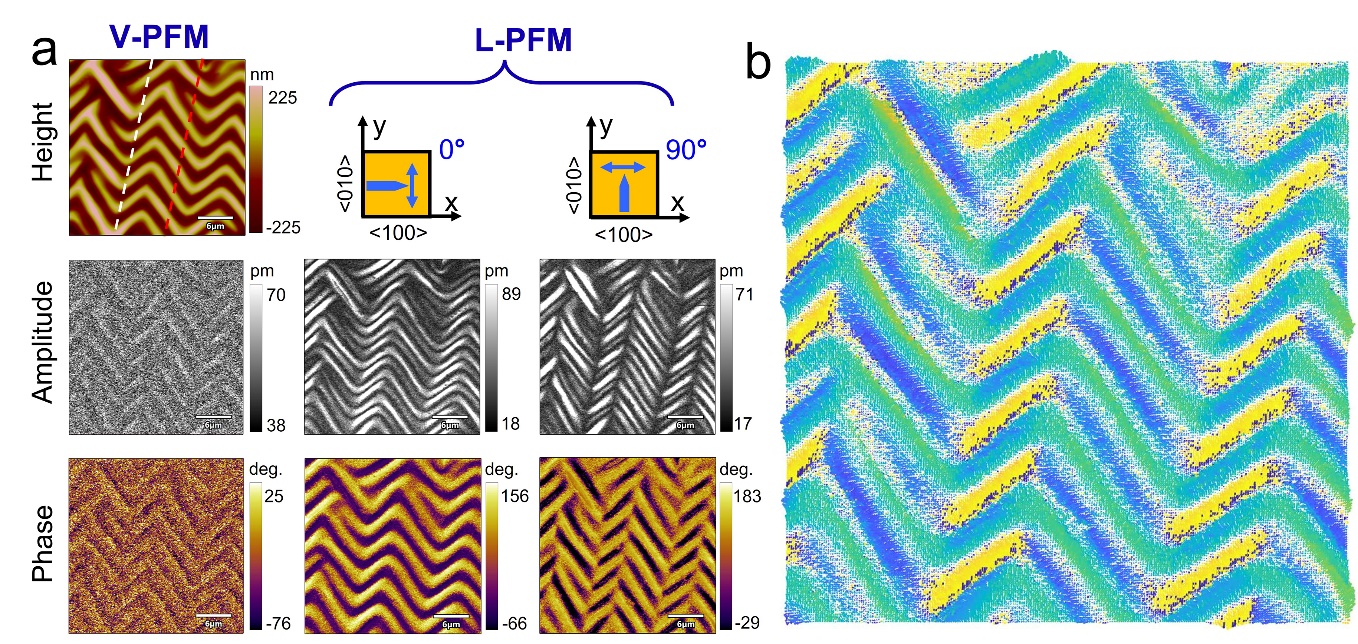


**Figure S11.** Domain structure of wrinkled BTO film in the same position as Figure 6 in the initial state. (a) The topography of the zigzag wrinkled BTO film and the amplitude and phase images of V-PFM and L-PFM (0° and 90°). (b) The two-dimensional polarization vector contours.

**10: Line profile of electrical switching behavior**


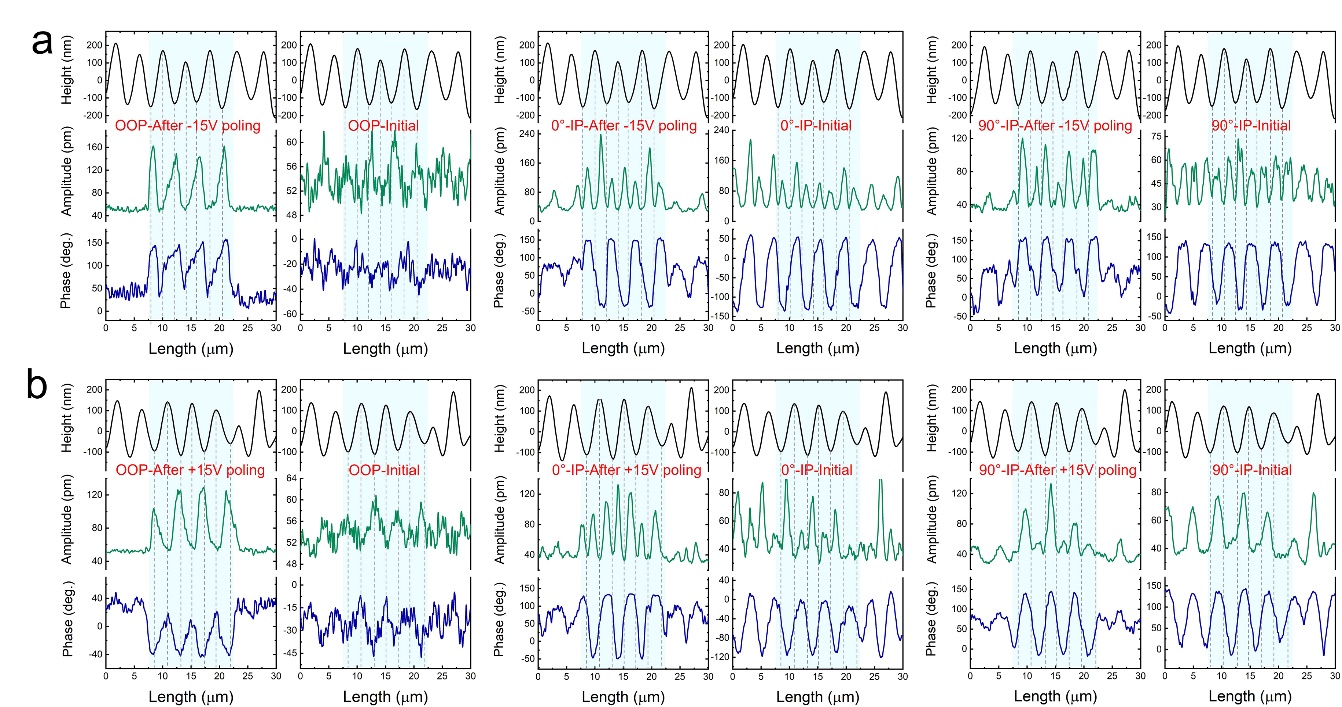


**Figure S12.** Line profiles of PFM in wrinkled BTO before and after applying voltage. The line profiles of the height, amplitude, and phase data (average value of 3 pixels) along the white (a) and red (b) dotted lines in Figures 5a and S7a.

**11: Retention of domain switching**


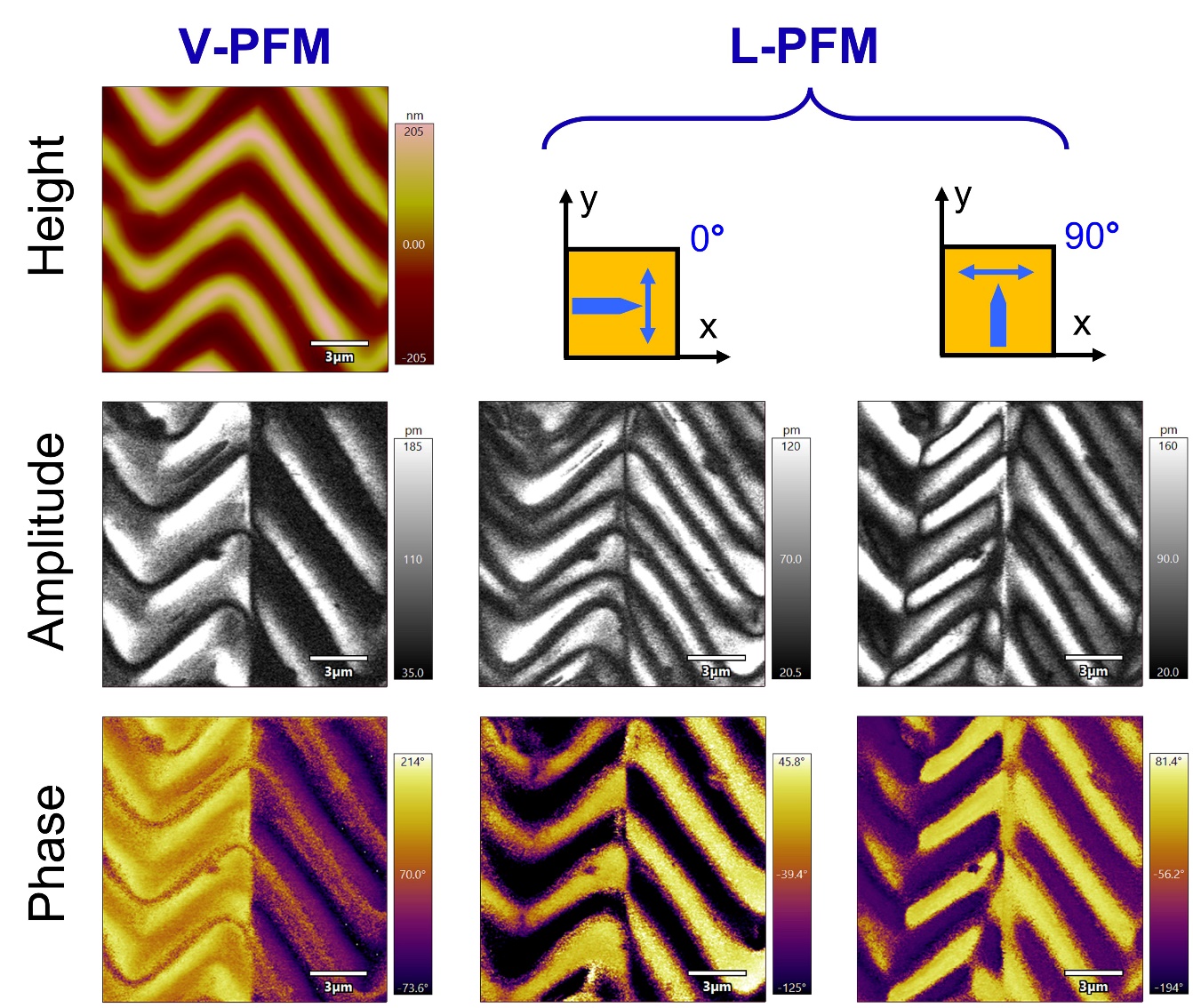


**Figure S13.** Topography, V-PFM, and L-PFM (0° and 90°) images of 15×15 µm^2^ area after writing voltage for 12 hours.
